# Programmable Invisible Photonic Patterns with Rapid Response Based on Two-Dimensional Colloidal Crystals

**DOI:** 10.3390/polym13121926

**Published:** 2021-06-10

**Authors:** Naiyu Liu, Zhikun Zheng, Dingshan Yu, Wei Hong, Hailu Liu, Xudong Chen

**Affiliations:** 1 Key Laboratory for Polymeric Composite and Functional Materials of Ministry of Education, School of Chemistry, Sun Yat-sen University, Guangzhou 510275, China; liuny6@mail2.sysu.edu.cn (N.L.); zhengzhikun@mail.sysu.edu.cn (Z.Z.); yudings@mail.sysu.edu.cn (D.Y.); hongwei9@mail.sysu.edu.cn (W.H.); 2 Guangdong Biomaterials Engineering Technology Research Center, Institute of Bioengineering, Guangdong Academy of Sciences, Guangzhou 510316, China

**Keywords:** anticounterfeiting, photonic crystals, structural color

## Abstract

The development of invisible patterns via programmable patterning can lead to promising applications in optical encryption. This study reports a facile method for building responsive photonic crystal patterns. Commercially printed patterns were used as a mask to induce invisible patterns revealed by wetting. The masked areas exhibit different swelling kinetics, leading to strong structural colors in the masked area and transparent features in the unmasked area. The contrast could disappear through different wetting behavior, providing a unique and reversible wetting feature. This programmable printing is expected to become an environmentally friendly technique for scalable invisible optical anti-counterfeiting technology.

## 1. Introduction

Photonic crystals have been extensively developed over the past few decades due to their effective light modulation and provide promising applications in diverse domains, such as anti-counterfeiting technologies [1,2], photocatalysis [3,4], decorations [5], and chemical and biological sensing [6,7]. Photonic crystal patterning enables the construction of high-performance devices over conventional photonic crystal films [8]. Patterning includes three methods. (1) Ink writing involves 3D printing with a colloidal suspension [9], or photonic precursors [10], as well as 2D patterning with a colloidal suspension [11], or solvents/salts/reactive monomers [12,13,14,15]. (2) Photomasking controls the degree of the reaction or crosslinking in ultraviolet curing systems, providing different patterns [16,17,18,19]. (3) Selective etching enables a top-down pattern modification of the formed photonic crystal, which can elaborately design, regulate, and control the micro- or nano-structure of the photonic crystal [20,21,22].

The development of invisible patterning based on photonic crystals offers potential design flexibility for optical encryption due to multiple and complex responsive strategies such as mechanochromic [23,24,25], magnetic [26,27,28], polarization-controlled [29], and wetting-controlled encryption [30,31,32]. In general, invisible patterning by photonic crystals depends on the shapes generated by the patterned field and increased contrast on sites developed by printing or photomasks stimulated by external signals. It is still challenging to realize rapidly responsive invisible structural color materials via scalable and programmable methods.

Herein, we report a programmable method to achieve wetting-responsive invisible patterns based on two-dimensional colloidal crystals. Large-scale two-dimensional colloidal crystals obtained by gas-liquid interfacial self-assembly [33,34] were used. To achieve invisible patterns, SiO_2_ (*n* = 1.46) [35], poly(hydroxyethyl methacrylate) (*n* = 1.45) and poly(ethylene glycol diacrylate) (*n* = 1.47), possessing similar refractive indices, were chosen to build photonic crystal films. A partially translucent mask printed on a plastic film was used to control the degree of polymerization. As the polymerization degree controls the swelling process, invisible patterns were observed upon water immersion. An increased difference in refractive index between the medium and photonic crystal gave rise to stronger Bragg’s diffraction light intensity in the masked area, and patterns were viewable in this area. The surface morphology was studied in different areas during the solvent response to explore the impact of solvent infiltration. To verify, Debye rings were measured to support this conclusion. Overall, this investigation offers a new solution for programmable photonic crystal anti-counterfeiting and a theoretical baseline for wetting-responsive photonic crystal patterning.

## 2. Materials and Methods

### 2.1. Materials

Monodisperse SiO_2_ spheres with diameters of 550 nm and 1 μm were supported by BaseLine Chromtech Research Centre. 2-hydroxyethyl methacrylate (HEMA), poly(ethylene glycol) diacrylate PEGDA (*M*_n_ ~ 700), trimethylolpropane triacrylate (TMPTA), and 2-hydroxy-2-methylpropiophenone were purchased from Macklin Biochemical Technology Co., Ltd., Shanghai, China. Other solvents and polypropylene (PP) films were supplied by local suppliers. All materials were used without further purification.

### 2.2. Preparation of Two-Dimensional Photonic Crystals

**Figure 1:**
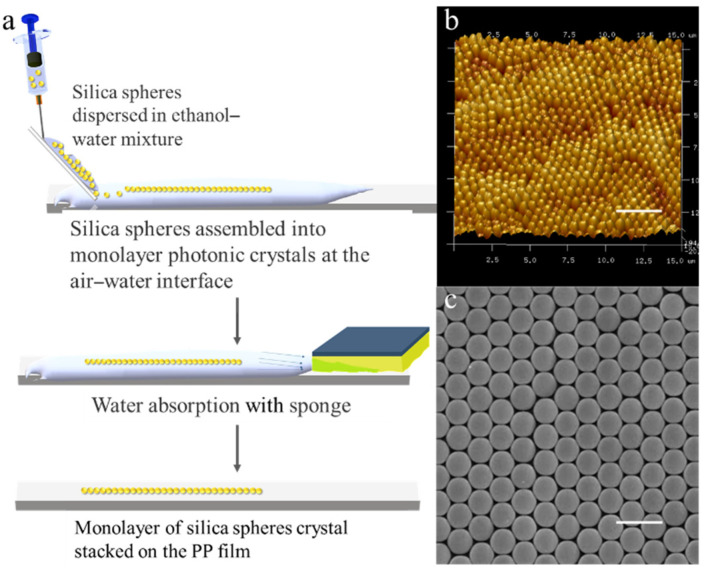
(**a**) Schematic of the monolayer crystalline silica spheres stacked on the PP film and the obtained AFM (**b**) and SEM (**c**) images (D = 550 nm) (Scale bars in Figure 1b,c are 3 μm and 1 μm, respectively).

For the preparation of two-dimensional photonic crystals, the gas-liquid self-assembly method was adopted. A layer of deionized water was spread on the PP plastic film with the size of an A4 paper (water contact angle: 88.0°). A glass sheet (50 mm × 16 mm × 0.18 mm) was immersed in water and fixed at 45°. A syringe was used to squeeze and drop an ethanol–water solution of silica with a mass fraction of 2.5 wt % and a particle size of 550 nm (volume ratio, ethanol:water = 1:1). Silica microspheres were evenly spread on the whole water surface and kept for 5 min, and a sponge was used to sop up water on the edge of the water quickly. They were dried in natural air for 2 h and introduced into a drying oven kept at 60 °C for 1 h to obtain extensive two-dimensional photonic crystals (Figure S1). The details on the preparation of two-dimensional photonic crystals are shown in Figure 1a.

### 2.3. Preparation of HEMA-PEGDA-Photonic Crystal Composite

**Figure 2:**
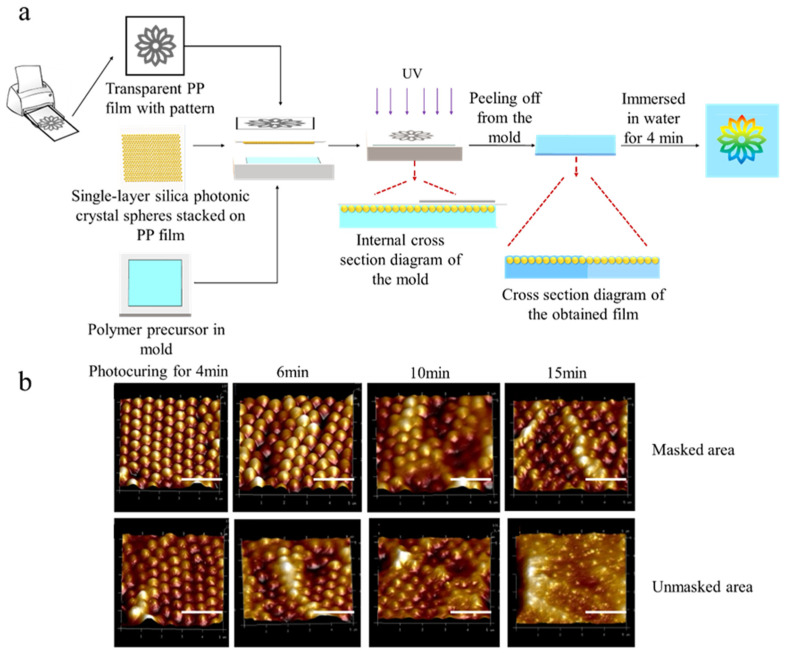
(**a**) Schematic shows the preparation of responsive photonic crystal film and (**b**) the AFM images of the obtained film with different photocuring times (scale bars in Figure 2b are 2 μm).

HEMA (300 μm), PEGDA (700 μm), TMPTA (10 μm) and 2-hydroxy-2-methylpropiophenone (10 μm) were pipetted sequentially and then mixed and transferred to a rectangular polytetrafluoroethylene mold (2 cm × 2 cm × 0.1 cm). Tweezers were then employed to place a photonic crystal plastic film (2.5 cm × 2.5 cm) on the mold. A mask was placed on the plastic and then introduced into an ultraviolet curing box (UV CURER KW-4AC, 365 nm). Unless otherwise specified, the polymerization time was 4 min, and the width of the mask was 0.3 cm. The details on the preparation of the HEMA-PEGDA-photonic crystal composite are shown in Figure 2a.

### 2.4. Characterization

Surface topography of obtained monolayer crystalline silica spheres stacked on the PP film were performed using Scanning Electron Microscope (S-4800, HITACHI, Ltd., Tokyo, Japan). Surface topography, roughness, and Young’s modulus of the photonic crystal composite surface were performed using Atomic Force Microscope (Dimension FastScan, BRUKER, Bremen, Germany) in the air or liquid phase. Doped Si tips (MPP-21100-10, BRUKER, Germany) (L = 225 μm; W = 35 μm; T = 3 μm), a cantilever elastic constant of 3 N/m and a resonant frequency of 75 kHz were used in all the measurements. Reflectance spectra were measured using fiber spectrophotometer (USB2000+, Ocean Optics Asia Co., Ltd., Shanghai, China.) coupled with a tungsten halogen light source (DH-2000-BAL, Ocean Optics), and the two fibers independently probed the incident and reflected light. Static contact angles were measured using a contact angle meter (DSA 100, Krüss, Frankfurt am Main, Germany). Droplets with 3 μL of deionized water were used in all the measurements. Five different positions were examined on each sample, and the final contact angle was computed by averaging the five obtained values. XPS data were obtained on Thermo ESCALAB 250, US, instrument with a monochromatized Al Kα line source (200 W). The binding energies were referenced to the Si2p peak at 101.8 eV.

## 3. Results and Discussion

### 3.1. Monolayer Two-Dimensional Photonic Crystals

Monodispersed microspheres were self-assembled on the gas–liquid interfaces to achieve two-dimensional colloidal crystals with the particle sizes of 550 nm deposited on a polypropylene (PP) plastic film (Figure 1a and Figure S1 in Supplementary Materials). As shown in the AFM and SEM analyses (Figure 1b,c), silica photonic crystals prepared by the gas–liquid interface assemblies were regularly arranged, and microspheres featuring a hexagonal, densely packed arrangement are shown on the PP plastic substrate. The formation of silica photonic crystals was driven by surface tension and capillary forces [36].

### 3.2. Physicochemical Properties and Morphological Characterization of Regions with Different Photocuring Degrees

The preparation of the photonic crystal film is shown in Figure 2a. Hydroxyethyl methacrylate and poly(ethylene glycol) diacrylate with refractive indices approximately equivalent to silica microspheres were used as polymer precursors simultaneously. To better understand the changes in the responsive photonic crystal films during photocuring, AFM tests were conducted to investigate the impact of different photocuring reaction times on the surface morphology of the film. As demonstrated in Figure 2b, the silica arrangement in the masked area is more regular than in the unmasked area. The spatial order of microspheres decreased with an increase in the photocuring reaction time. When the photocuring reaction time increased to 15 min, the polymer surface became smooth and flat, and the microspheres were almost embedded fully. Changes in the film morphology obtained during photocuring are schematically shown in Figure 2b.

The AFM feature map in Figure 2b was measured to obtain the roughness of different areas of the film (Table S1 in Supplementary Materials). The roughness of both masked and unmasked areas decreased with increasing reaction time, and when subjected to the same photocuring reaction time, the masked area of the film was rougher than the unmasked area. When the photocuring reaction time was increased continuously, the roughness of the masked area was approximately equal to the unmasked area. This indicates that both the reduction in photocuring reaction time and the use of a mask can assist in controlling the exposed area of silica microspheres so that the unevenness on the upper surface of the film is more obvious. On the same film, the one-step method cannot control the reaction time so that the degree of reaction is controlled; however, it is easy and convenient to control the degree of reaction through masking.

**Figure 3:**
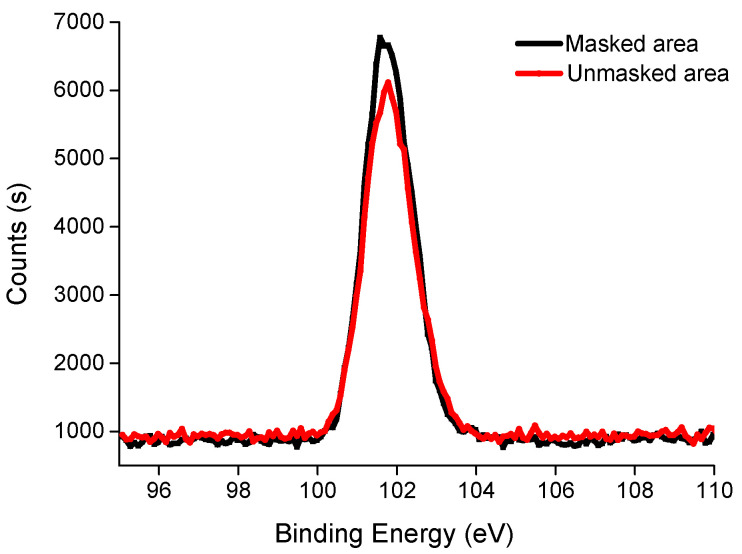
XPS spectrum on the surfaces of the masked and unmasked areas.

When liquid polymer precursor contacts two-dimensional silica microspheres, the liquid rose along the gap with capillary action. Zhan et al. [35] used two-dimensional photonic crystals and elastomers to obtain inverse opal structured material. Different thermal reaction temperatures and times affected the depth of aperture, and as the degree of reaction increased, it was easier to embed microspheres into the polymer. The chemical composition of the surface in different areas was experimentally compared by detecting the binding energy of silica (Figure 3), where atomic content in Si2p in the masked and unmasked areas was 4.51% and 3.49%, respectively. The Si2p element was available on the polymer surface, and the content in the masked area was higher than the unmasked area. This result agrees with the conclusion reported by Zhan et al., where the degree of reaction in the unmasked area was high and it was shown to be easier for the polymer to embed silica microspheres. When the polymerization photocuring reaction time was 3 min, a certain color appears in the air in the masked area. The exposed area of the silica microspheres in the masked area was larger, and Bragg’s diffraction light was enhanced. However, to guarantee the degree of polymerization, in most experimental samples, the photocuring reaction time was 4 min, and no luster was found in the masked area on the resulting sample surface.

### 3.3. Patterning of Responsive Photonic Crystal Films and the Reflectance Spectra

**Figure 4:**
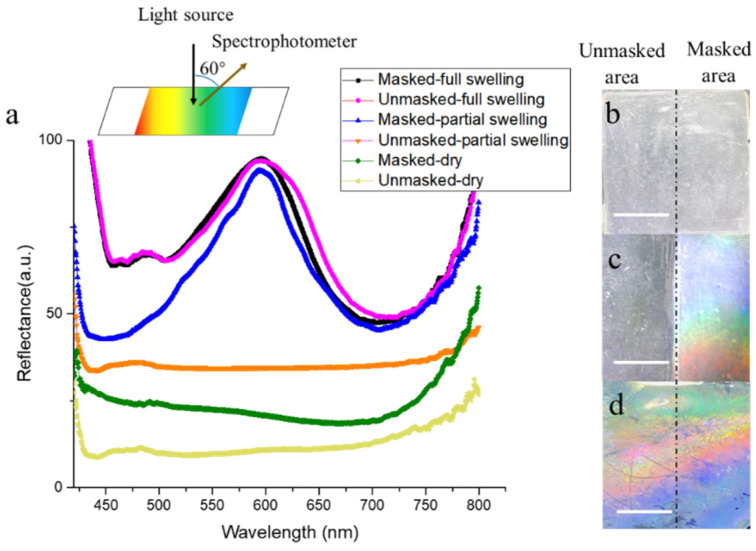
(**a**) Spectrum of different areas in different states; optical images of a half-masked film in three conditions: dry (**b**), partial swelling (**c**), and full swelling (**d**) (scale bars are 1 cm).

In controlling the degree of polymerization by the mask, a new responsive photonic crystal pattern could be realized by water. As shown in Figure 4b–d, a half-masked film does not exhibit any color in the dry state while the masked area turned colorful via water immersion for 4 min. Interestingly, when the film was immersed in water for 1.5 h, the whole film became colorful. The optical fiber spectroscopy (Figure 4a) recorded spectral differences between the two areas in Figure 4b–d.

**Figure 5:**
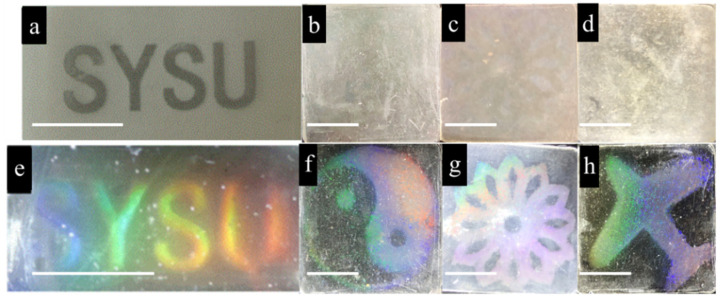
Optical images of (**a**) gray-scale picture on transparent PP film that serves as a mask; (**b**–**d**) film in a dry state; (**e**–**h**) film immersed in the water (scale bars: 1 cm).

Meanwhile, the laser printer’s gray-scale maps printed on transparent PP films (Figure 5a) served as masks to print patterns on the film. Different types of patterns could hide in the dry state (Figure 5b–d) and developed after wetting, such as the letters SYSU, the Tai Chi symbol, a flower, and an airplane (Figure 5e,f). This demonstrates that this method allows a wide range of patterns could be obtained quickly and easily, from simple letters to complex images.

A fiber optic spectrometer was used to examine the reflectance spectra of film in different states. Two mutually orthogonal fibers were independently probed by the incident and reflected light. The incident angle and reflected angle were manually set to 0° and 32°, respectively, and the incident angle and reflected angle were changed simultaneously by rotating the angle of the sample stage.

**Figure 6:**
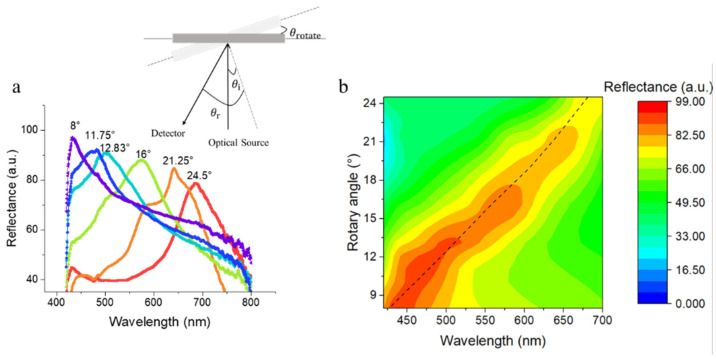
(**a**) Reflectance spectrum of the masked area in a wet state for the rotation angle from 8° to 24.5°, and the illustration in the upper right corner is the schematic diagram of the spectral testing device. (**b**) Angle-resolved reflectance spectra of the masked area in a wet state for the rotation angle from 8° to 24.5°. The black dashed curve denotes the calculated reflectance maxima of the photonic stop band.

Based on the combined Bragg’s and Snell’s laws, the relationship of the parameters and reflection wavelengths can be obtained according to light diffraction and refraction theories [37,38] (Equation (1)).

(1) $$m\lambda =n_{\mathrm{eff}}d\left({\sin\theta }_{i}+{\sin\theta }_{r}\right),$$

(2) $$n_{\mathrm{eff}}^{2}=\sum n_{i}^{2}\mathrm{Vi},\;$$

where m is an integer connected with an anti-reflective coating, λ is the wavelength of the reflected light, θ_i_ is the incident angle, θ_r_ is the reflected angle, n_eff_ is the average refraction coefficient, n_i_ is the refraction coefficient of the photonic crystal’s ingredients, and V_i_ is the volume fraction of the photonic crystal’s ingredients. According to Equation (1), changing θ_i_ or θ_r_ induces changes in λ. Figure 6a shows the reflectance spectra of films with the rotation angle of the sample stage varied from 8° to 24.5°, in which the diffraction maxima red-shifted with the increasing rotation angle. The overall trends agree with the calculated results based on Equation (1), denoted by the black dashed curve in Figure 6b.

Reusability is an important aspect for evaluating the actual application value of anti-counterfeiting materials. Figure S2 (in Supplementary Materials) shows the reflection spectra of the masked area after nine cycles of wetting and drying. It displays the wet masked area with obvious diffraction peaks, while the dry masked area does not exhibit any diffraction peaks (Figure S2a). In addition, the wet films over nine cycles maintained a stable diffraction peak intensity, and the dry samples also had a certain improvement in the reflection strength in the next four cycles (Figure S2b). This could be because a part of the unpolymerized monomers in the film could separate from the film during multiple infiltrations, leading to enhanced intensity of the diffraction peak and overall reflectivity. Since the responsive color caused by water is reversible, and its reusability is high, the cyclically utilized film devices are more convenient to use and save costs.

The simple one-step method based on a mask with partial masking is mainly used to control the degree of reaction. It is expected to become a new anti-counterfeiting printing technology. Therefore, the impact of mask width on the geometric accuracy of patterning is very important. Gray-scale lines printed by a laser printer were used to make plastic films with different widths (Figure S3a in Supplementary Materials), and they were taken as templates to obtain films with different stripe patterns (Figure S3b in Supplementary Materials). When the width of the plastic mask was less than 1 mm, the pattern colors became very weak.

### 3.4. Impact of Water on the Micro-Morphology of Responsive Photonic Crystal Patterning

**Figure 7:**
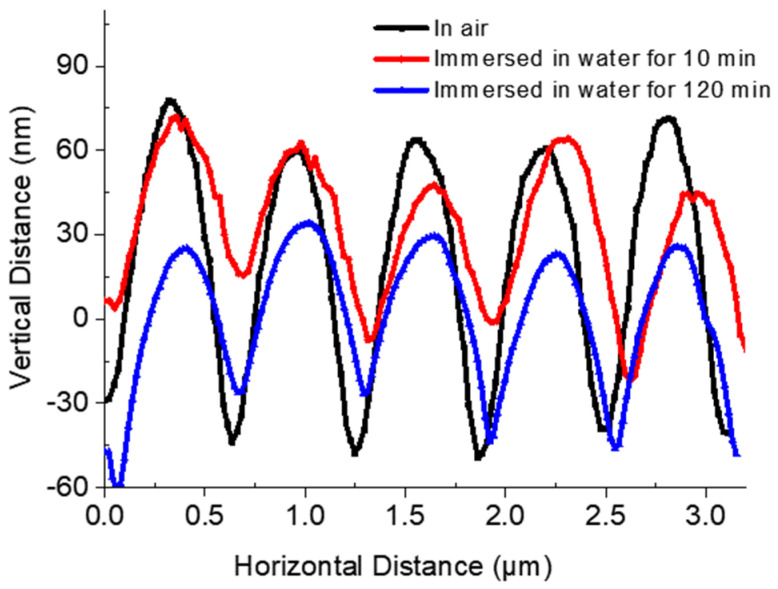
AFM images of the height profile scanned across the line about five silica microspheres of the masked area in the air, immersed in water for 10 min, and immersed in water for 120 min.

Subsequently, AFM was used to observe the impact of the medium on the micro-morphology of responsive photonic crystal patterns, to obtain more useful conclusions. The impact of water on the apparent morphology of the pattern was studied (Figure S4 in Supplementary Materials), and five microspheres were taken from the masked area, and the morphological height maps in the air and water were compared (Figure 7).

In a self-made drilled mold, deionized water was injected until it was just overflowing. It was surrounded by a double-faced adhesive tape so that the lower surface of the film in the masked area was closely stuck on the mold. The impact on the surface morphology of the film after immersing the lower surface into deionized water was measured. After immersing for 10 min, the average surface height was 65.40 nm. Immersion was continued for 120 min, and the average surface height was 66.43 nm, while the average surface height of film in the masked area in the air was 106.69 nm. The immersion of the film’s lower surface in the water decreased the average surface weight, and the immersion time exhibits less impact on the height.

**Figure 8:**
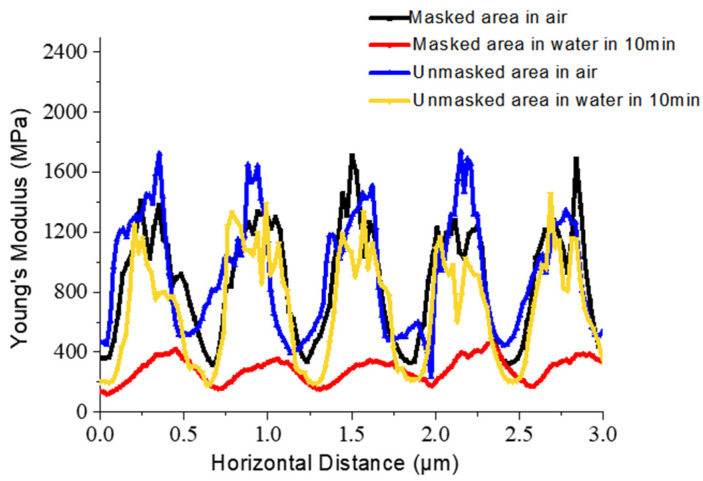
The AFM DMT modulus profile scanned across a line about five silica microspheres of masked area in the air, masked area in water, unmasked area in the air, and unmasked area in water.

As indicated by AFM testing (Figure 8), Young’s modulus of the masked and unmasked areas in the air was very high and exceeded 1.5 GPa. The Young’s modulus of the masked area was higher than that of the unmasked area, suggesting a larger exposed area of silica, which agrees with the results of XPS testing. After immersing into the water, Young’s modulus of the masked area significantly decreased compared to the unmasked area and was only tens of MPa.

A decrease in Young’s modulus may be due to the decrease in the mechanical properties of media surrounding the microspheres caused by water infiltration. Subsequently, the contact angle (CA) of water was measured in different areas of the film to compare wettability between the masked and unmasked areas (Table 1). The contact angle (CA) of the water was measured in different areas of the film to compare the wettability between the masked and unmasked areas (Table 1). The CA of lower surfaces in two areas had a significantly increasing tendency with an increase in photocuring time, while that of the upper surface was slightly increased. The higher CA of the lower surface compared to the same photocuring time of the upper surface is due to the presence of silica nanospheres. Focusing on the upper surface in two areas, the CA of the masked area was lower than that of the unmasked area due to the complete embedding of silica microspheres, which agrees with the AFM results (Figure 2b).

**Figure 9:**
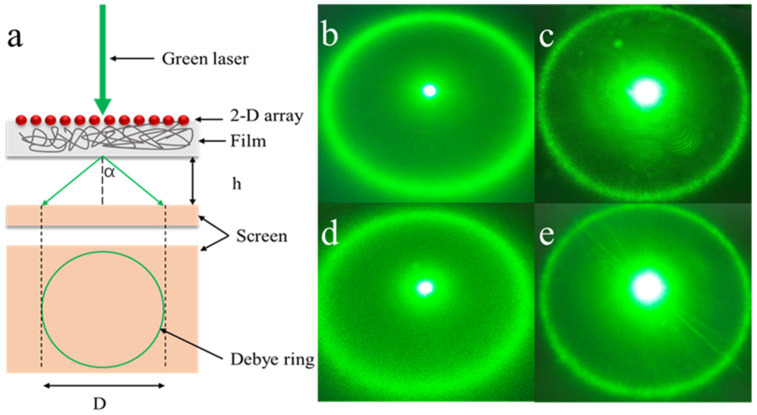
(**a**) Schematic diagram of the Debye ring measurement; optical Debye ring images of (**b**) unmasked area in a dry state and (**c**) unmasked area by immersing in water for 2 min; (**d**,**e**) masked area under the same state as (**a**,**b**).

To examine whether the difference in the difficulty of swelling leads to the appearance of the pattern or not, Debye ring diffraction measurements of thin films were carried out to monitor particle spacing. For this measurement, silica spheres (1 μm) were used to prepare the thin films [39]. Figure 9a schematically shows that in the Debye ring diffraction measurement geometry, the first-order diffraction angle, *α*, depends on the particle spacing.

(3) $$\sin\alpha =2\lambda_{\mathrm{laser}}/\left(\sqrt{3}d\right),$$

where α is the interior angle of the Debye diffraction ring, λ_laser_ is the laser wavelength, and d is the particle spacing. The diffraction angle α can be determined from the Debye diffraction ring diameter (Equation (4)).

(4) $$\alpha =\tan^{-1}\left(\frac{D}{2h}\right),$$

We could monitor particle spacing by measuring the Debye ring diameter. Figure 9b–e exhibit Debye rings of the masked and unmasked areas under drying and water immersion for 4 min, respectively. By measuring D and h, the particle cycles of the two areas in the two states were calculated and are shown in Table 2. In the dry state, both the particle spacing of the masked and unmasked areas are about 1180 nm, but after immersing in water for 4 min, the particle spacing of the two areas increased to a certain extent. The above results further prove that the masked area swelled more quickly under the same short-term immersion conditions than the unmasked area.

### 3.5. The Mechanism of Pattern Generation

**Figure 10:**
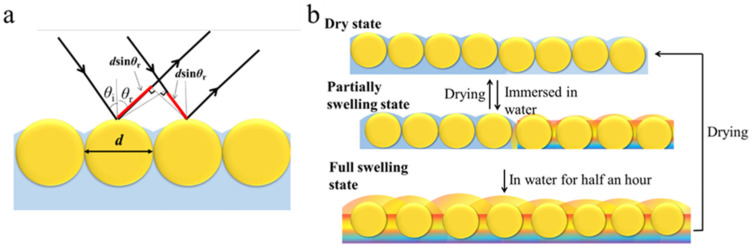
(**a**) Reflection path of the incident light on 2D photonic crystal; (**b**) swelling–deswelling process of photonic crystal pattern.

Photonic crystals refer to periodic arrangements of different dielectric media in one-dimensional, two-dimensional, or three-dimensional space. When light propagates in these media, it follows the theory of light diffraction and refraction (Figure 10a) and generates Bragg’s diffraction based on combined Bragg’s and Snell’s laws. According to Equations (1) and (2), the changes in the position of the photonic bandgap are associated with the lattice parameter of the photonic crystal, average refraction coefficient, and the refractive index of the photonic crystal medium, in addition to the nature of the raw material [40].

The AFM and XPS data indicate that the higher the degree of the photocuring reaction, the more complete the embedding of the silica microspheres, confirming a higher polymer content between the silica microspheres in the masked area. Therefore, the surface of the masked area is rougher, and the arrangement of the internal silica microspheres is more compact. Additionally, the average refraction coefficient of the media is similar to that of silica, causing unobservable Bragg’s diffraction of the dry film.

However, when the film was immersed in water for a short time, the situation is different. The images and testing results of the masked area show intense diffracted light once it was immersed in water, which comes from the decreased average refraction coefficient of the media while partially swelling. However, these changes are localized in the masked area as the degree of polymerization of the covered area is lower than that of the uncovered area, making it easier for water molecules to penetrate [35]. The difference in the degree of aggregation causes the masked area to swell faster than the unmasked area, as proved by the testing results. However, long-term immersion could eliminate the differences in swelling speed in the two areas and turn the entire film colorful. In this situation, the whole film is completely swollen so that the refraction coefficient of the media and silica microspheres is no longer approximate and induces Bragg’s diffraction light intensely. Meanwhile, drying could eliminate color, achieving the film’s reusability. A schematic diagram of the swelling–deswelling process is shown in Figure 10b. The reported method of building quick response patterns from this study will be a new guideline in anti-counterfeiting.

## 4. Conclusions

A partially translucent mask was used to control the degree of photocuring reaction and study the physicochemical properties and morphological differences in the surfaces of the masked and unmasked areas. The obtained results show that lowering photocuring degree can help control the exposure extent of silica microspheres by reducing the photocuring time or using a mask. By immersing in water, the infiltration of water induces a decrease in the medium’s refractive index, causing intense Bragg’s diffraction. Using a mask can lower the photocuring degree and localize its effects within some areas, realizing the responsive pattern. Since the pattern visualization originates from the swelling of the medium, desiccating the film can hide the pattern again. This swelling–deswelling process is reversible, endowing responsive photonic crystal films with high reusability. This programmable method can be conveniently employed to obtain nano anti-counterfeiting material, where hidden patterns can be visualized using only a drop of water, providing a new wettability-sensitive anti-counterfeiting technology.

## Figures and Tables

**Table 1:** The contact angle vs. photocuring time for the upper and lower surface of the film in the masked and unmasked areas.

Area	Contact Angle of Upper Surface (°)			Contact Angle of Lower Surface (°)		
	3 min	4 min	6 min	3 min	4 min	6 min
Masked	50.6	54	53.6	58	60.2	88.2
Unmasked	68.1	75.5	75.1	89.6	93.5	92.3

**Table 2:** Particle spacing of different areas in under different states.

	Dry (nm)	Partial Swelling (nm)
Unmasked area	1183.89	1288.23
Masked area	1178.79	1349.55

## Data Availability

Data available on request from corresponding author.

## Supplementary Materials

The following are available online at https://www.mdpi.com/article/10.3390/polym13121926/s1. Figure S1: Ultra-fast fabrication of large-area 2D colloidal photonic crystals assembly at the air-water interface, Table S1: The roughness vs. photocuring time in the masked and unmasked areas, Figure S2: (a) Reflection spectra of the masked area of nine cycles of wetting and drying; (b) Reflectance versus cycle times for nine cycles of wetting and drying, Figure S3: The optical precision vs. mask width in the masked area, Figure S4: AFM images of the surface topography in the masked area.
