# Supplementary material for: Programmable Invisible Photonic Patterns with Rapid Response Based on Two-Dimensional Colloidal Crystals
Source: Polymers (Basel). 2021 Jun 10;13(12):1926. doi: 10.3390/polym13121926 (PMC8226874; doi:10.3390/polym13121926)
Supplement: Supplementary file 1 [file polymers-13-01926-s001.zip › polymers-1195774-supplementary.pdf]

polymers

MDPI

Article

Programmable Invisible Photonic Patterns with Rapid Response Based on Two-Dimensional Colloidal Crystals

Naiyu Liu [1], Zhikun Zheng [1], Dingshan Yu [1], Wei Hong [1], Hailu Liu [2,*] and Xudong Chen [1,*]

[1] Key Laboratory for Polymeric Composite and Functional Materials of Ministry of Education, School of Chemistry, Sun Yat-sen University, Guangzhou 510275, China; liuny6@mail2.sysu.edu.cn (N.L.); zhengzhikun@mail.sysu.edu.cn (Z.Z.); yudings@mail.sysu.edu.cn (D.Y.); hongwei9@mail.sysu.edu.cn (W.H.)
[2] Guangdong Biomaterials Engineering Technology Research Center, Institute of Bioengineering, Guangdong Academy of Sciences, Guangzhou 510316, China
* Correspondence: shirley1418@126.com (H.L.); cescxd@mail.sysu.edu.cn (X.C.)

Citation: Liu, N.; Zheng, Z.; Yu, D.; Hong, W.; Liu, H.; Chen, X. Programmable Invisible Photonic Patterns with Rapid Response Based on Two-dimensional Colloidal Crystals. *Polymers* **2021**, *13*, x. https://doi.org/10.3390/xxxxx

Academic Editor: José Manuel Gaspar Martinho

Received: 8 April 2021
Accepted: 21 May 2021
Published: date

Publisher's Note: MDPI stays neutral with regard to jurisdictional claims in published maps and institutional affiliations.

Copyright: © 2021 by the authors. Licensee MDPI, Basel, Switzerland. This article is an open access article distributed under the terms and conditions of the Creative Commons Attribution (CC BY) license (http://creativecommons.org/licenses/by/4.0/).

Supporting Informations

Table S1. The roughness vs. photocuring time in the masked and unmasked areas (Rq is referred to as the root-mean-square roughness).

Area	Roughness (nm)	Light Curing Time			
		4Min	6Min	10Min	15Min
**Masked**	Rq	41.1	38	34.7	24.6
Unmasked	Rq	31.3	30.3	23.3	25.8

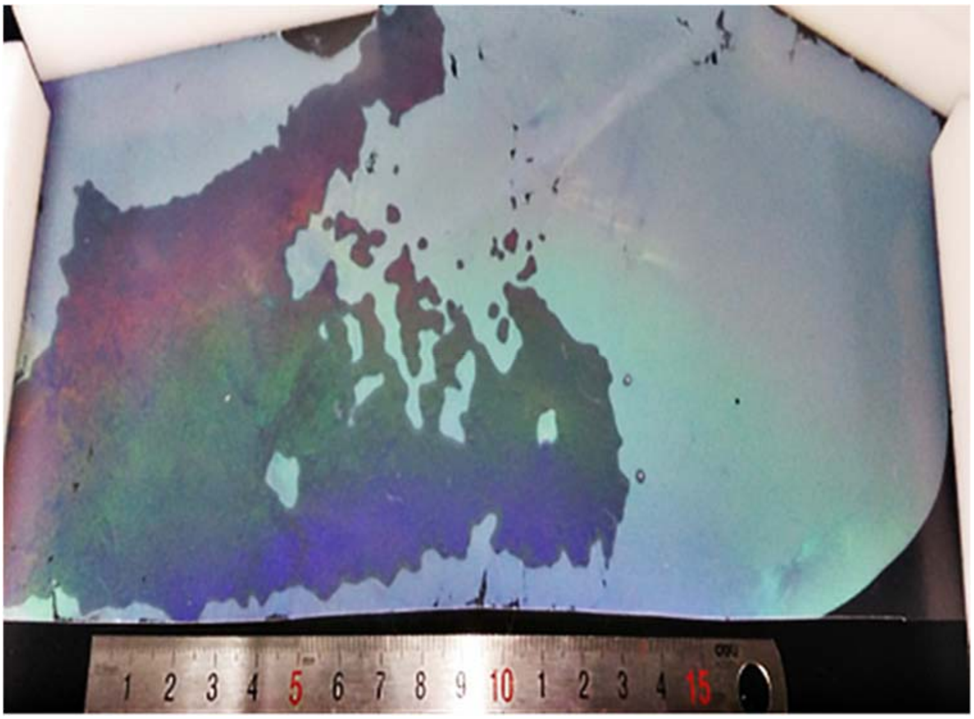

Figure S1. Ultra-fast fabrication of large-area 2D colloidal photonic crystals assembly at the air-water interface.

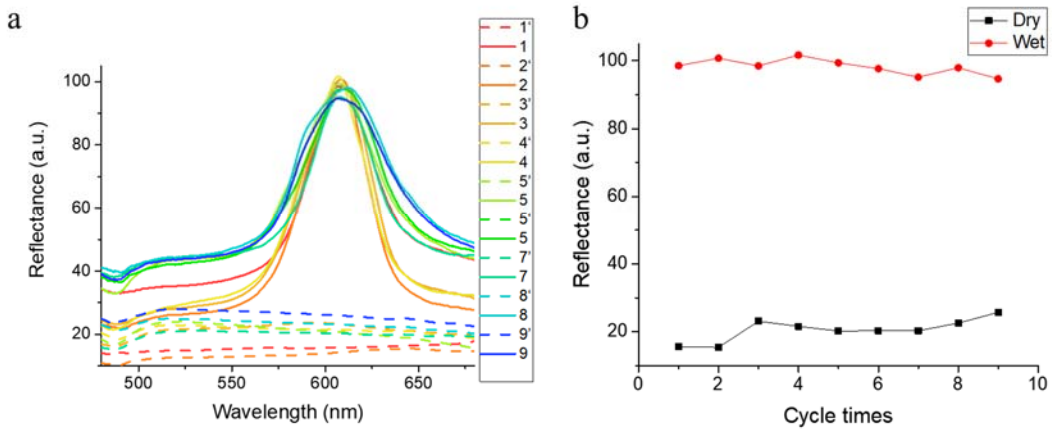

Figure S2. (**a**) Reflection spectra of the masked area of nine cycles of wetting and drying; (**b**) Reflectance versus cycle times for nine cycles of wetting and drying.

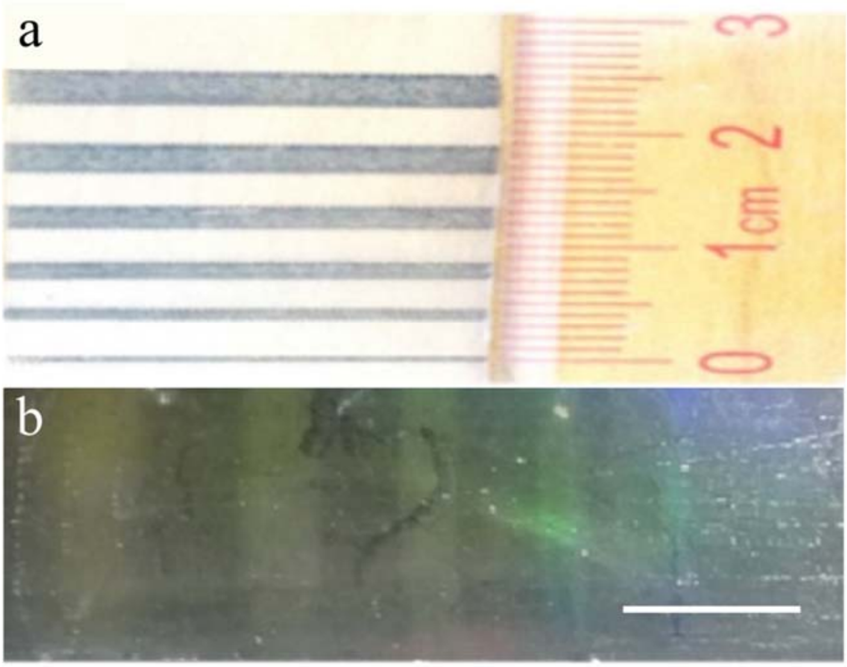

Figure S3. The optical precision vs. mask width in the masked area, Figure S4. AFM images of the surface topography in the masked area.

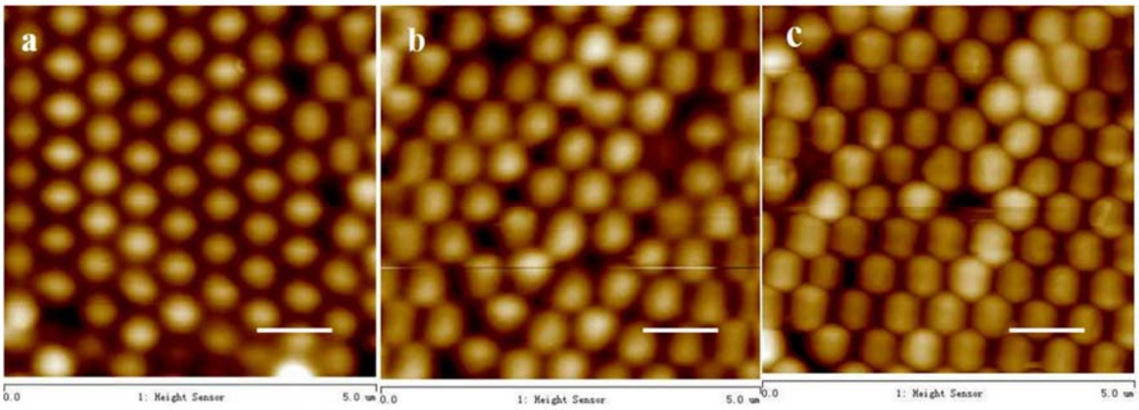

Figure S4. AFM images of the surface topography in the masked area. (**a**) in air; (**b**) immersed in water for 10 min; (**c**) immersed in water for 120 min (The scale bars are 1 μm).
